# Barriers to Therapeutic Use of Hydroxyurea for Sickle Cell Disease in Nigeria: A Cross-Sectional Survey

**DOI:** 10.3389/fgene.2021.765958

**Published:** 2022-01-19

**Authors:** Emmanuel Chide Okocha, Joyce Gyamfi, Nessa Ryan, Oluwatoyin Babalola, Eno-Abasi Etuk, Reuben Chianumba, Maxwell Nwegbu, Hezekiah Isa, Anazoeze Jude Madu, Samuel Adegoke, Uche Nnebe-Agumandu, Biobele Brown, Emmanuel Peprah, Obiageli E. Nnodu

**Affiliations:** ^1^ Haematology Department, Nnamdi Azikiwe University Teaching Hospital, Nnewi, Nigeria; ^2^ Global Health Program and Department of Social and Behavioral Sciences, NYU School of Global Public Health, New York, NY, United States; ^3^ Department of Biotechnology, Chrisland University, Abeokuta, Nigeria; ^4^ Centre of Excellence for Sickle Cell Disease Research and Training (CESRTA), University of Abuja, Abuja, Nigeria; ^5^ Department of Haematology and Immunology, College of Medicine, University of Nigeria, Ituku-Ozalla Campus, Enugu, Nigeria; ^6^ Department of Paediatrics and Child Health, Obafemi Awolowo University, Ile-Ife, Nigeria; ^7^ Department of Paediatrics, College of Medicine, University of Ibadan and University College Hospital, Ibadan, Nigeria

**Keywords:** hydroxyurea, sickle cell diasease, Nigeria, adoption, health care workers

## Abstract

**Background:** Sickle cell disease, the inherited blood disorder characterized by anemia, severe pain and other vaso-occlusive complications, acute chest syndrome, disproportionate hospitalization, and early mortality, has significant financial, social, and psychosocial impacts and drains individuals, families, and health systems globally. Hydroxyurea could improve the health of the 300,000 individuals born each year with sickle cell disease in sub-Saharan Africa; however, challenges to adoption and adherence persist. This study assessed the barriers to therapeutic use of hydroxyurea for sickle cell disease within the Nigerian healthcare system, specifically from the level of the patient, provider, and health system.

**Methods:** We used purposive sampling to recruit participants from 13 regions in Nigeria. A cross-sectional survey was administered to physicians (*n* = 70), nurses or counselors (*n* = 17), and patients or their caregivers (*n* = 33) at 13 health centers. Findings were mapped onto the appropriate Consolidated Framework for Implementation Research (CFIR) domains.

**Results:** This study was able to identify factors that mapped onto the inner setting, outer setting, and characteristics of individuals domains of CFIR. The majority of physicians (74.3%) prescribe hydroxyurea, and half stated hydroxyurea is the standard of care. Among clinicians, barriers included limited knowledge of the drug, as well as low self-efficacy to prescribe among physicians and to counsel among nurses; perceived side effects; perceived patient preference for traditional medicine; cost for patient and expense of accompanying laboratory monitoring; and limited availability of the drug and equipment for laboratory monitoring. Among patients and caregivers, barriers included lack of knowledge; perceived side effects; cost; religious beliefs of disease causation; and lack of pediatric formulation.

**Conclusions:** Findings suggest that patient, provider, and health systems-level interventions are needed to improve hydroxyurea uptake among providers and adherence among patients with sickle cell disease in Nigeria. Interventions such as patient education, provider training, and policy change could address the disproportionate burden of sickle cell disease in sub-Saharan Africa and thus improve health equity.

## Background

Sickle cell disease (SCD), the inherited blood disorder characterized by anemia, severe pain and other vaso-occlusive complications, acute chest syndrome, disproportionate hospitalization, and early mortality, has significant financial, social, and psychosocial impacts and drains individuals, families, and health systems. Currently, it is projected that more than 300,000 individuals are born annually with SCD in sub-Saharan Africa (SSA) (Ohene-Frempong et al., 2008; Brown et al., 2010; Piel et al., 2013). SCD is a progressively debilitating and a chronic multi-organ disease with a 30–50% incidence of disability and unemployment, as well as the leading cause of stroke in children and adolescents (Corbacioglu, 2016). Hydroxyurea is efficacious in improving hematological parameters of sickle cell patients by promoting the production of younger erythrocytes with higher hemoglobin F (HbF) content and less tendency to polymerize, thereby reducing sickling with resultant increase in hematocrit (Hassan et al., 1995; Youssry et al., 2017; Ofakunrin et al., 2020). These ameliorating effects have translated into improved patient outcomes, including reduced rate of vaso-occlusive crisis, blood transfusions, hospitalizations, and incidence of acute chest syndrome, as well as improved organ function and overall survival (S. Charache et al., 1995; Youssry et al., 2017; Ofakunrin et al., 2020). Although hydroxyurea was approved for SCD management in adult patients by the US FDA in 1998 (Ault, 1998), for pediatric patients in 2017, and specifically for children within low and middle income countries (LMICs) in 2018, (Tshilolo et al., 2019) challenges to its routine use in LMICs persist (Gyamfi et al., 2021a; Gyamfi et al., 2021b).

Nigeria has the highest burden of SCD worldwide and yet, the use of hydroxyurea among SCD patients in Nigeria is very low. Galadanci and colleagues (2014) (Galadanci et al., 2014) found that only eight of 18 SCD specialist health institutions studied in Nigeria prescribed hydroxyurea to their patients, and within those institutions, only 5–33% of their patients were on hydroxyurea (Adegoke et al., 2015; Esezobor et al., 2016; Adewoyin et al., 2017; Adeyemo et al., 2019). Although, the safety and adverse side effects of hydroxyurea—the only cost-effective pharmacotherapeutic compound that can be effectively delivered in SSA—have been documented, (Adewoyin et al., 2017; Adeyemo et al., 2019; Aliyu et al., 2007; Charache et al., 1995; S.; Charache et al., 1995) and the overall clinical effectiveness of hydroxyurea to ameliorate SCD severity is well established in Nigeria and other African countries, (Tshilolo et al., 2019) its adoption is still low. Therefore, we examined the barriers preventing adequate uptake of hydroxyurea, including prescription and adherence, for therapeutic use among SCD patients in the Nigerian healthcare system.

## Methods

### Conceptual Framework

Damschroder’s Consolidated Framework for Implementation Research (CFIR) (Damschroder et al., 2009), was used as a theoretical framework to guide the conceptualization of the results and understand the barriers preventing adequate uptake of hydroxyurea, including prescription and adherence, for therapeutic use among SCD patients in the Nigerian healthcare system. Specifically because this was a cross-sectional study, we were only able to focus on the following three CFIR domains: inner setting (e.g., health systems characteristics and resources – availability of HU), outer setting (e.g., patients adherence to HU) and characteristics of individuals involved (e.g., providers’ prescription practice).

### Design

A cross-sectional study was conducted in 13 health facilities across Nigeria using an anonymous questionnaire among clinical providers, patients, and caregivers. We aimed to examine the barriers to therapeutic use of hydroxyurea among SCD patients in the Nigerian healthcare system.

### Ethical Approval and Consent to Participate

Ethical approval was secured from the National health research ethics committee of Nigeria (NHREC). NHREC Protocol Number NHREC/01/01/2007- 21/11/2017. NHREC Approval Number NHREC/01/01/2007-03/11/2019C. All adult participants provided verbal consent and parents consented on behalf of their children.

### Setting, Sampling, and Recruitment

Using purposive sampling, participants were recruited in-person from healthcare centers across 13 different regions of Nigeria, including Oyo, Osun state, Lagos, Kano, Kaduna, Gombe, FCT, Enugu, Edo, Ebonyi, Delta State, Benue, Anambra. As the survey was anonymous and did not collect identifiable data, verbal consent was sought from in-person respondents or inferred when the participant moved on from the first page of the online survey.

### Data Collection and Analysis

The questionnaire was developed after a focused discussion within a collaborative group of local clinicians and US-based implementation scientists, comprised of individuals with expertise in local context or implementation research. Potential participants were sent the survey *via* Survey Monkey where internet service was available, or by hard copy if internet service was not available (and responses were later transferred online). The questionnaire was administered to 120 respondents in secondary and tertiary healthcare centers where SCD patients receive care, including physicians, nurses or counselors that treat SCD patients, as well as SCD patients or their caregivers. Respondents, depending on level of education and availability of internet services, self-administered the questionnaire on paper or online, or were assisted by a trained data collector. Data collection took place during July 2019.

The structured questionnaire consisted of two parts: socio-demographic characteristics and challenges to therapeutic use of hydroxyurea. Among physicians, drug potency and availability, accompanying laboratory services, patient adherence, and personal objections to use were ascertained. Among nurses, counselors, patients, and caregivers, questions were modified for their respective roles. With nurses or counselors, for example, questions asked about issues affecting ability to counsel the patient appropriately, while questions for patients and caregivers included how religion, availability of alternative therapies, and personal awareness affected hydroxyurea use.

Data analysis was conducted using SPSS version 20.0 (SPSS Inc., Chicago, IL, United States). Descriptive statistics were performed and relevant frequencies and proportions calculated.

## Results

### Characteristics of Study Sample

A total of 120 respondents completed the questionnaire, including physicians (*n* = 70), nurses or counselors (*n* = 17), and patients or their caregivers (*n* = 33). The sample was 50.8% female, with an average age of 38.5 ± 13.3 years. A majority of the participants were recruited from tertiary (85.7%) and public (97.5%) health facilities (Table 1).

**TABLE 1:** Characteristics of study population and various clinical study facilities across Nigeria, 2019 (*N* = 120).

Variables		Frequency	%
Age in years (*n* = 105)			
	≤20	14	13.3
	21–30	11	10.5
	31–40	33	31.4
	41–50	27	25.7
	>50	20	19.1
Gender (*n* = 120)			
	Male	59	49.2
	Female	61	50.8
Occupation (*n* = 120)			

	Doctor	70	58.3
	Nurse	17	14.2
	Civil Servant	6	5.0
	Teacher	2	1.7
	Business	5	4.2
	Technician	1	0.8
	Youth Corper	1	0.8
	Student	17	14.2
	Unemployed	1	0.8
Marital status (*n* = 120)			
	Married	87	72.5
	Single	33	27.5
Level of education (*n* = 118)			
	None	1	0.9
	Primary	4	3.4
	Secondary	15	12.7
	Undergraduate	22	18.6
	Postgraduate	76	64.4
History of hydroxyurea use by patients (*n* = 33)			
	Yes	16	48.5
	Yes, but stopped using HU	4	12.1
	No	3	9.1
	Missing	10	30.3
Level of health care facility (*n* = 119)			
	Secondary	17	14.3
	Tertiary	102	85.7
Type of health care facility (*n* = 119)			
	Public	116	97.5
	Private	3	2.5

### Characteristics of Individuals

#### Uptake: Provider Prescription Practice

Among physicians, three out of 4 (74.3%) reported they prescribed hydroxyurea to their patients (Table 2). While 61.4% of physicians knew that SCD clinical management guidelines recommend therapeutic use of hydroxyurea, about half stated that hydroxyurea is the standard of care. Though a few (4.3%) physicians reported they were unaware of the medication, 17.6% of the nurses or counselors stated that they were unaware of hydroxyurea.

**TABLE 2:** Clinician knowledge and practice regarding hydroxyurea at clinical facilities across Nigeria, 2019 (*N* = 87).

Questions	Responses
	Yes *n* (%)
Physicians (*n* = 70)	
Hydroxyurea (HU) prescription and knowledge	
Prescribe HU to patients	52 (74.3)
I don’t know about HU	3 (4.3)
Have no formal training on HU prescription	19 (27.1)
Have formal training on how to counsel patients about HU use	20 (28.6)
Not confident in providing HU to my patients	5 (7.1)
There is no data that shows it will work in our clime	4 (5.7)
It is the standard of care for SCD	39 (55.7)
The guidelines for care of SCD recommend its use	43 (27.1)
Patients are using HU	28 (40.0)
HU is expensive, my patients cannot afford it.	18 (25.7)
HU is a cytotoxic drug and can cause cancer	13 (18.6)
HU has other side effects	47 (67.1)
Not convinced of the safety of HU for treatment of SCD	3 (4.3)
Patients’ compliance	
HU prescribed, but patient compliance is poor	15 (21.4)
Patients complain about the side effects of HU	16 (22.9)
HU is available, but not in the formulation children can use	2 (2.9)
Drug potency	
Prescribed HU and found it ineffective	1 (1.4)
Prescribed HU and found it quite effective	36 (51.4)
Availability of drug/lab services to monitor	
HU is available at my site but the supply is limited	26 (37.1)
HU is not available at my site	6 (8.6)
My patients cannot access it from another location	3 (4.3)
My patients can access it from another location	29 (41.4)
The hospital I work in does not have the laboratory facilities to monitor the use of this drug	6 (8.6)
Nurses or counselors (*n* = 17)	
Personal knowledge	
I do not know about HU	3 (17.6)
I have no formal training on how to counsel patients about HU	12 (70.6)
Cost	
Patients complain about funds to purchase HU	4 (23.5)
Our patients are too poor and cannot afford this drug	4 (23.5)
Our patients usually commence using HU, but stop because they cannot afford the cost of investigations needed to monitor this drug	2 (11.8)
Availability	
HU is good, but our patients can’t find it to buy	8 (47.1)
Compliance	
Our patients do not like taking HU because of side effects	1 (5.9)
Patients usually commence taking this drug, but stop because of side effects	4 (23.5)
Our patients usually do well on the drug and compliance is excellent	8 (47.1)
Prescription/Counseling issues	
Our doctors do not prescribe HU	1 (5.9)
Our counseling about this drug is ineffective with our patients	0 (0.0)
I do not have formal training about counselling patients about HU	3 (17.6)
Personal reasons	
The relatives of the patients discourage the use of HU	0 (0)
Patient are not motivated to use HU	2 (11.8)

### Inner Setting

#### Barriers to Hydroxyurea Prescription

Various barriers to prescribing hydroxyurea for SCD management were reported by clinicians, including lack of knowledge and self-efficacy, perceived affordability for the patient, perceived side effects, and perceived patient preference for traditional medicine. Some (27.1%) of the physicians reported no formal training on prescribing hydroxyurea, along with the majority (70.6%) of nurses or counselors who reported no formal training on counseling patients on hydroxyurea use. A few (7.1%) physicians were not confident about prescribing hydroxyurea to their patients. Some (25.7%) physicians stated that hydroxyurea is expensive and could not be afforded by their patients. The majority (67.1%) of physicians believed that hydroxyurea has side effects; 18.6% believed hydroxyurea is cytotoxic and carcinogenic (Table 2). Two doctors agreed that their patients preferred traditional medicines to western drugs, and one doctor was of the opinion that the use of hydroxyurea would make no difference to the health of patients.

### Barriers to Availability of Hydroxyurea and Laboratory Monitoring

Physicians reported various health system barriers regarding availability of hydroxyurea and laboratory monitoring. About one in three physicians stated that hydroxyurea was available in their center with limited supply, while one in 10 stated that it was not available. Four in 10 physicians also stated their patients could access the drug from another location. Six doctors admitted that the hospitals where they work do not have the laboratory facilities to monitor the use of the drug. Also, 12% of the patients stated that the accompanying investigations needed to monitor the drug are too expensive and unaffordable. Only one in three patients stated that hydroxyurea was available at their health center. Five caregivers stated that the drug was not available in the formulation suitable for their child.

### Outer Setting

#### Uptake: Patient Adherence

For physicians who prescribed the medication, 61.5% stated that most of their patients were on hydroxyurea, while the remaining 38.4% of physicians stated only a few of their patients were on hydroxyurea. One in five physicians reported that patients complain about the side effects of hydroxyurea. Of the nurses, one in four agreed that patients discontinue use due to side effects and one in ten noted that patients are not motivated to use hydroxyurea. Of the patients and caretakers, only 30% could afford to buy the drug (Table 3). Six patients saw no need to continue the use of hydroxyurea for religious reasons, since by faith they felt they had been healed.

**TABLE 3:** Factors influencing hydroxyurea use among sickle cell disease patients as reported by the patients or their caregivers at various clinical facilities across Nigeria, 2019: *N* = 33 (pediatric (*n* = 7), adults (*n* = 26)).

Questions	Responses
	Yes *n* (%)
Cost	
HU is very expensive, and l cannot afford it	4 (12.1)
I can afford to buy the drug	10 (30.3)
The investigations needed to monitor this drug are too expensive. l cannot afford them	4 (12.1)
Availability	
The drug is available at my health center	11 (33.3)
It is not available in the formulation in which l/my child needs it	5 (15.1)
Faith	
By faith l know l am healed, so no need to continue this drug	6 (18.1)
Alternative therapy	
I do not want to take this drug, because l am waiting to get the opportunity to do a bone marrow transplant	0 (0)
Compliance	
l/my ward vomits whenever this drug is taken, so l discontinued it	1 (3.0)
My/my wards skin color was changing so l discontinued it	0 (0)
Drug potency	
l/my ward had a crisis, while on this drug, so l feel it is not effective. l discontinued it	1 (3.0)
This drug is very good and has relieved a lot of my symptoms that’s why l continue to take it	10 (30.3)
Personal awareness	
My doctor/medical counselor said that the drug has ability to cause cancer so I cannot take it	0 (0)
The medical staff don’t really know the long term effects of this drug, it has not been used in SCD for long enough. I prefer to wait till they are sure	0 (0)

### Barriers to Patient Adherence

Various barriers to patient adherence to therapeutic use of hydroxyurea were reported by clinicians and patients. Among the physicians, one in five noted poor patient adherence after prescription; the same number indicated that their patients reported side effects. Two physicians agreed that hydroxyurea is available, but not in the formulation children can use. For nurses, 47.1% reported that their patients did well on hydroxyurea and that adherence was good. Half of the prescribing physicians found hydroxyurea effective while only one disagreed. Among patients, 30% reported that hydroxyurea is good and had relieved a lot of their symptoms, while one patient found the drug ineffective and discontinued its use.

## Discussion

Hydroxyurea, the first line drug of treatment for sickle cell disease, is under-utilized in Nigerian patients, as is likely the case in various other LMICs. This study was able to identify factors that mapped onto the inner setting, outer setting, and characteristics of individuals domains of CFIR and found that, although the majority of physicians (74.1%) prescribed hydroxyurea to patients, much fewer (40%) report their patients actually use hydroxyurea. This is an improvement on what was observed almost a decade ago in Nigeria (Galadanci et al., 2014); however, there is still a gap between knowledge of this evidence-based practice and its routine implementation.

This work identifies various reasons for this “know-do” gap, which should be examined in future research. Physicians and nurses alike reported they lacked training on hydroxyurea prescription and patient counseling, respectively. The fear of side effects, cost, and unavailability of hydroxyurea and accompanying laboratory monitoring were major barriers to hydroxyurea uptake and patient adherence. Indeed, others have examined how toxicity and safety issues, (Adegoke et al., 2015; Adewoyin et al., 2017; Tshilolo et al., 2019) side effects, patient adherence, and lack of a national guideline for the use of hydroxyurea have discouraged prescription by physicians in Nigeria (Adewoyin et al., 2017; Adeyemo et al., 2019; Aliyu et al., 2007; Charache et al., 1995; S.; Charache et al., 1995). Moreover, side effects such as skin rashes, dark patches on skin, vomiting, and dizziness have been observed in some hydroxyurea users in Nigeria (Adewoyin et al., 2017) and this may further inhibit its use. Previous research has examined concerns about differential host response to hydroxyurea among children with SCD in relation to malnutrition, infections, infestations, and issues of feasibility of lifelong use, considering Nigeria’s socioeconomic circumstances and weak healthcare system (Tshilolo et al., 2019).

About half of the patients in this study were taking hydroxyurea, but some stopped due to side effects. Side effects such as hair loss, nausea, neutropenia, and oligospermia are reversible; and often may not reappear when hydroxyurea is stopped and restarted at a lower dose. Therefore, a consent procedure involving patients and their caregivers, emphasizing benefits and clarifying the possibility of reversible side effects, may help to improve hydroxyurea uptake (Smith et al., 2019). Additionally, patients are supposed to be monitored for HbF induction and full blood count parameters and, if undesirable effects such as very low blood cell counts (neutropenia) are observed, medication is discontinued temporarily. A blood count is done 1–2 weeks after to check for recovery, and treatment continued at the same or reduced dose after recovery. Proper education of patients and caregivers about the reversibility of side effects during the recovery window might encourage more patients to continue hydroxyurea uptake until the desired effects are observed. Another concern especially in children is a higher susceptibility to infection due to neutropenia. However, clinical trials by Opoko et al., 2017 showed no difference in susceptibility to malaria in children placed on hydroxyurea compared to those not placed on hydroxyurea. Nonetheless, Tshilolo and colleagues (Tshilolo et al., 2019), have shown a lower rate of malaria cases in children placed on hydroxyurea as compared to those not placed on hydroxyurea.

The unavailability of equipment and reagents to monitor the patients may also pose a barrier to therapeutic use of hydroxyurea for SCD in Nigeria. Equipment to quantify hemoglobin variants and monitor the HbF levels is expensive, so few centers in Nigeria have them (Galadanci et al., 2014). The cost of clinical monitoring is also expensive and unaffordable for many patients. The issue of cost and availability of the drug as raised by the participants of the study is an important one that should also be addressed. A pack of 100 tablets of hydroxyurea is N12, 800 (∼$40), which is quite expensive for most Nigerians. The cost of hydroxyurea should be subsidized, the drug made readily available in all sickle cell clinics and registered pharmaceutical outlets, and the paediatric formulation should be made readily available.

Although there are various strengths to this study, there are some limitations to address. The small sample size and the cross-sectional nature of the study may limit generalizability of the findings. Similar studies in Nigeria with a larger sample size and better national coverage may help to uncover additional factors that limit hydroxyurea utility in Nigeria. Also, future research studies should consider an intervention which will allow for examination of additional domains of CFIR including intervention characteristics (e.g., evidence strength and quality, adaptability), and the process of implementation (e.g., planning and executing an intervention protocol). Despite the study limitations, this study sheds light on the challenges to hydroxyurea adoption in Nigeria, a resource limited setting.

## Conclusion

In Nigeria, hydroxyurea uptake is limited by provider prescription practices and patient adherence. This work identified the barriers and recommends interventions targeting an increase in provider, patient, and caregiver knowledge regarding the benefits of hydroxyurea. Particularly, formal training of haematologists and nurses/counselors on hydroxyurea prescription, as well as monitoring and counseling challenges should be addressed across health centers to ensure that SCD patients can get the drug when it is prescribed in low-resource settings.

## Data Availability

The original contributions presented in the study are included in the article/Supplementary Material, further inquiries can be directed to the corresponding authors.
